# Height, weight and BMI percentiles and nutritional status relative to the international growth references among Pakistani school-aged children

**DOI:** 10.1186/1471-2431-12-31

**Published:** 2012-03-19

**Authors:** Muhammad Umair Mushtaq, Sibgha Gull, Komal Mushtaq, Hussain Muhammad Abdullah, Usman Khurshid, Ubeera Shahid, Mushtaq Ahmad Shad, Javed Akram

**Affiliations:** 1 Ubeera Memorial Research Society, Allama Iqbal Medical College, Lahore 54000, Pakistan; 2 District Health Office Nankana Sahib, Punjab Department of Health, Nankana, Sahib 39100, Pakistan

## Abstract

**Background:**

Child growth is internationally recognized as an important indicator of nutritional status and health in populations. This study was aimed to compare age- and gender-specific height, weight and BMI percentiles and nutritional status relative to the international growth references among Pakistani school-aged children.

**Methods:**

A population-based study was conducted with a multistage cluster sample of 1860 children aged five to twelve years in Lahore, Pakistan. Smoothed height, weight and BMI percentile curves were obtained and comparison was made with the World Health Organization 2007 (WHO) and United States' Centers for Disease Control and Prevention 2000 (USCDC) references. Over- and under-nutrition were defined according to the WHO and USCDC references, and the International Obesity Task Force (IOTF) cut-offs. Simple descriptive statistics were used and statistical significance was considered at P < 0.05.

**Results:**

Height, weight and BMI percentiles increased with age among both boys and girls, and both had approximately the same height and a lower weight and BMI as compared to the WHO and USCDC references. Mean differences from zero for height-, weight- and BMI-for-age z score values relative to the WHO and USCDC references were significant (P < 0.001). Means of height-for-age (present study: 0.00, WHO: -0.19, USCDC: -0.24), weight-for-age (present study: 0.00, WHO: -0.22, USCDC: -0.48) and BMI-for-age (present study: 0.00, WHO: -0.32, USCDC: -0.53) z score values relative to the WHO reference were closer to zero and the present study as compared to the USCDC reference. Mean differences between weight-for-age (0.19, 95% CI 0.10-0.30) and BMI-for-age (0.21, 95% CI 0.11-0.30) z scores relative to the WHO and USCDC references were significant. Over-nutrition estimates were higher (P < 0.001) by the WHO reference as compared to the USCDC reference (17% vs. 15% overweight and 7.5% vs. 4% obesity) while underweight and thinness/wasting were lower (P < 0.001) by the WHO reference as compared to the USCDC reference (7% vs. 12% underweight and 10% vs. 13% thinness). Significantly lower overweight (8%) and obesity (5%) prevalence and higher thinness grade one prevalence (19%) was seen with use of the IOTF cut-offs as compared to the WHO and USCDC references. Mean difference between height-for-age z scores and difference in stunting prevalence relative to the WHO and USCDC references was not significant.

**Conclusion:**

Pakistani school-aged children significantly differed from the WHO and USCDC references. However, z score means relative to the WHO reference were closer to zero and the present study as compared to the USCDC reference. Overweight and obesity were significantly higher while underweight and thinness/wasting were significantly lower relative to the WHO reference as compared to the USCDC reference and the IOTF cut-offs. New growth charts for Pakistani children based on a nationally representative sample should be developed. Nevertheless, shifting to use of the 2007 WHO child growth reference might have important implications for child health programs and primary care pediatric clinics.

## Background

Child growth is internationally recognized as an important indicator of nutritional status and health in populations [1,2]. Growth monitoring is an integral component of preventive and primary care pediatrics to evaluate individual children, and is a useful public health tool to assess child health status and economic development in the society [3,4]. Growth reference based on a nationally representative sample has not been developed for Pakistani children and Pakistan is one of the countries still using the 1977 National Center for Health Statistics (NCHS) reference for pediatric growth monitoring [5]. The NCHS dataset as an international growth reference has been challenged on serious technical grounds and its continued use is being discouraged [6-8]. Recently updated international height, weight, and BMI references for children and adolescents by the World Health Organization 2007 (WHO) and United States' Centers for Disease Control and Prevention 2000 (USCDC) are of interest as potentially useful for Pakistani children [9,10].

Interpretation of child growth in a population depends primarily on the growth reference used [11]. Literature lacks data on nutritional parameters and indices of nutritional status with reference to international growth references among Pakistani school-aged children. This study was aimed to compare age- and gender-specific height, weight and BMI percentiles and nutritional status relative to the international growth references among Pakistani school-aged children.

## Methods

### Design, setting and sample

A population-based cross-sectional study titled 'Nutritional Assessment among School-going Children in Lahore, Pakistan (NASCL)' was conducted among primary school children aged five to twelve years in 2009-2010. Lahore, a metropolis with multiethnic populations, is the capital of Pakistan's most populous province Punjab. It has a population of nine million including 2.5 million primary school children, and 81% of the population resides in the urban area [12].

A representative multistage cluster sample of 1860 children aged five to twelve years in twelve primary schools of City District Lahore was enrolled. In the first stage, stratified random sampling, based on population and educational system characteristics, was used to have proportionate representation of gender, area of residence and socioeconomic status (SES). The list of all the public and private primary schools in Lahore was provided by the Punjab Department of Education. The listed schools were stratified according to the geographic area and monthly fee structure of schools into the following four strata: a) urban with high SES (urban area and fee > 2500 PKR), b) urban with middle SES (urban area and fee = 1000-2500 PKR), c) urban with low SES (urban area and fee < 1000 PKR), and d) rural with low/disadvantaged SES (rural area and fee ~100 PKR or free). The former two strata included private (including public-private mix) schools and the later two strata included public schools. Three schools were selected at random from each stratum and contacted by the Departments of Education and Health to participate voluntarily in the study. If the school administration refused to participate, next school was selected randomly from the respective stratum. For each school, a list of all classes in five grades (one to five) was obtained and one class in each grade was selected at random. In this way, sixty classes, five from each school, were selected. For each of the selected classes, first thirty-one children on class attendance register, present on data collection day and aged five to twelve years, were included in the study. Children suffering with any known metabolic syndrome (e.g. Prader-Willi syndrome) and children not willing to participate in the study were excluded. Sample size was calculated using Epi Info 6.04 d (USCDC, 2004) with a confidence (1-α) of 95%, anticipated prevalence of 5% and margin of error of ± 1. The minimum sample size calculated was 1823 and a sample of 1860 was deemed sufficient.

### Data collection

The sampled schools were visited on pre-arranged dates in summer 2009. Twenty trained senior medical students including 10 males and 10 females, lead by the Principal Investigator, collected the data. Data collection activity in each school was completed in two working days and it took four weeks to complete the data collection. Health education of children and teachers was also carried out after data collection in the respective school. Analogue physician health scales, standardized before the examination, were used [13]. The instruments were checked and calibrated on a daily basis. Height measurement was in centimeters (cm) to the nearest 0.1 cm and weight was measured in kilogram (kg) to the nearest 0.5 kg with a range of 0-160 kg. The child was asked to stand relax, feet were placed together with heels, buttocks and shoulder blades against the stick and head was positioned in the Frankfurt horizontal plane. All measurements were taken in light summer school uniform without shoes during mornings or early afternoons. The measurements were tested for reliability (one-week test-retest). For each of the sampled classes, demographic information of all officially enrolled students was obtained before data collection, including gender and date of birth. Demographic information of students not found on official rosters but currently enrolled in that class was obtained from class teachers.

Quality control measures and good practices including training, pre-testing the processes and materials, field monitoring of data collection, logistics management and daily meetings of the study teams were ensured. Informed consent statement was printed on the form. Verbal informed consent for the child to participate in the study was taken from class teachers and school heads. As the study involved no invasive procedure, verbal informed consent was deemed sufficient. The study was approved by the Ethical Review Board of Allama Iqbal Medical College, Lahore. Permissions to conduct the study were granted by the Punjab Departments of Education and Health, and the sampled schools.

### Statistical analysis

Data were entered and analyzed by manual and computerized checking using SPSS version 18.0 (SPSS Inc. Chicago IL, United States, 2009). Age was calculated to the precise day by subtracting the date of birth from the date of examination. Smoothed percentile curves and z score values for height-, weight- and BMI-for-age relative to the present sample were obtained by the LMS method [14,15]. The z score values for height-, weight- and BMI-for-age relative to the WHO 2007 reference were calculated using WHO AnthroPlus [16,17]. The WHO 2007 reference does not provide weight reference values for children older than 10 years; therefore, z-score values for weight-for-age were calculated for five to ten years. The z score values relative to the USCDC 2000 reference were calculated by the SPSS files provided by the USCDC [18]. Overweight (> + 1SD BMI-for-age z score), obesity (> + 2SD BMI-for-age z score), thinness/wasting (< -2SD of BMI-for-age z score), underweight (< -2SD of weight-for-age z score) and stunting (< -2SD of height-for-age z score) were defined according to the WHO and USCDC references. Weight-for-age is inadequate indicator for monitoring child growth beyond pre-school years due to its inability to distinguish between relative height and body mass, therefore, BMI-for-age is recommended by the WHO and USCDC to assess thinness/wasting in school-aged children and adolescents [9,19]. Overweight, obesity and thinness were also defined using the International Obesity Task Force (IOTF) cut-offs [15,20]. Difference from zero for means of height-, weight- and BMI-for-age z scores, relative to the WHO and USCDC references and the present study, were calculated with Student's *t*-test. Differences in height-, weight- and BMI-for-age z score means between the WHO and USCDC references were compared by paired *t*-test. McNemar test was used to compare differences in prevalence of over- and under-nutrition by the WHO and USCDC references. Statistical significance was considered at P < 0.05 and all tests were two-sided.

## Results

The study included a sample of 1860 primary school children aged five to twelve years. The male-female ratio was 1.11 with 52.5% boys and 47.5% girls. The sample involved 20% children from each grade and 25% children from each area and SES stratum. Seventy-five percent children were urban and 25% were rural. The median age (range) was 8 (5-12) years. Mean and standard deviation (SD) for height, weight and BMI were 128.4 (11.4) cm, 26.9 (8.5) kg and 16.0 (3.0) kg/m^2 ^respectively (Table 1).

**Table 1:** Mean and standard deviation (SD) for height, weight and BMI of Pakistani primary school children aged five to twelve years (n = 1860)

Characteristics	n	Height (cm)	Weight (kg)	BMI (kg/m^2^)
**Boys (n = 977)**				

5 years (61-71 months)	84	113.7 (7.3)	19.9 (4.6)	15.2 (2.1)

6 years (72-83 months)	161	118.3 (5.9)	21.6 (5.0)	15.3 (2.8)

7 years (84-95 months)	160	122.9 (8.0)	23.5 (5.1)	15.5 (2.4)

8 years (96-107 months)	158	128.7 (7.6)	26.9 (5.9)	16.1 (2.5)

9 years (108-119 months)	161	134.2 (8.1)	29.7 (7.6)	16.4 (3.1)

10 years (120-131 months)	147	138.4 (8.0)	33.3 (9.5)	17.2 (3.5)

11 years (132-143 months)	69	138.6 (7.7)	31.8 (6.8)	16.5 (2.7)

12 years (144-155 months)	37	140.0 (8.3)	31.8 (7.3)	16.1 (2.3)

**Girls (n = 883)**				

5 years (61-71 months)	72	115.4 (7.3)	19.3 (3.2)	14.4 (1.5)

6 years (72-83 months)	143	119.1 (7.6)	21.0 (4.9)	14.7 (2.4)

7 years (84-95 months)	157	124.0 (6.3)	24.0 (5.5)	15.5 (2.7)

8 years (96-107 months)	159	128.1 (7.1)	26.4 (6.8)	15.9 (2.9)

9 years (108-119 months)	151	133.3 (7.8)	30.4 (8.2)	17.0 (3.5)

10 years (120-131 months)	120	138.4 (9.3)	33.3 (10.1)	17.2 (3.8)

11 years (132-143 months)	62	143.3 (9.6)	36.5 (11.0)	17.5 (3.7)

12 years (144-155 months)	19	146.0 (9.4)	36.4 (9.9)	16.9 (3.3)

**Figure 1:**
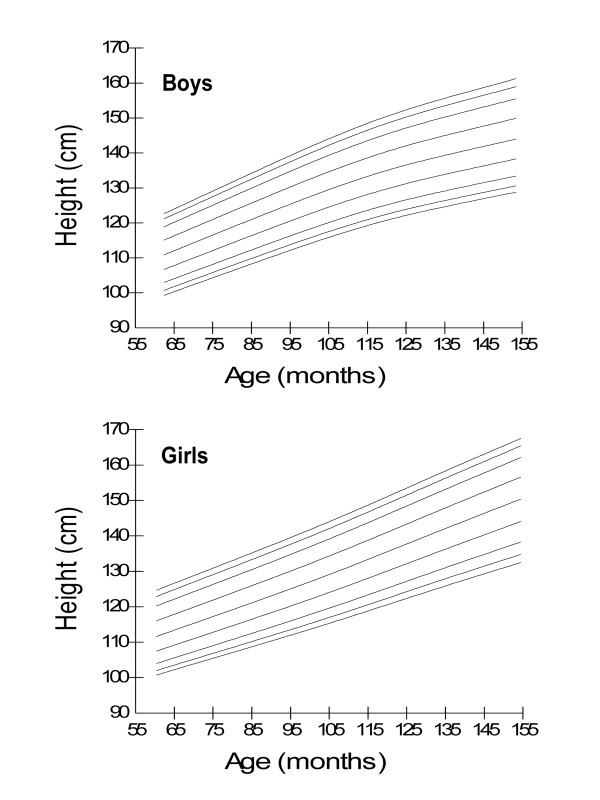
**Smoothed height percentile curves for Pakistani primary school boys (n = 997) and girls (n = 883) aged five to twelve years**. 5 years (61-71 months), 6 years (72-83 months), 7 years (84-95 months), 8 years (96-107 months), 9 years (108-119 months), 10 years (120-131 months), 11 years (132-143 months) and 12 years (144-155 months).

**Figure 2:**
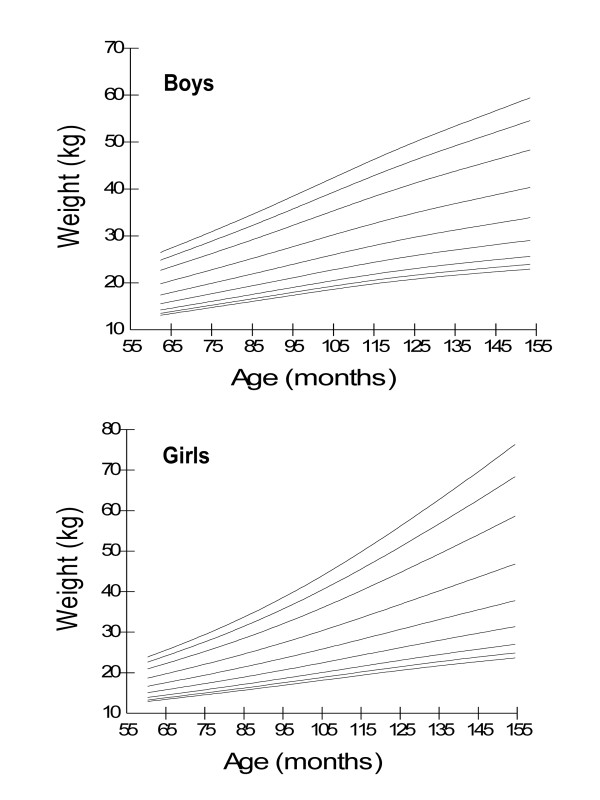
**Smoothed weight percentile curves for Pakistani primary school boys (n = 997) and girls (n = 883) aged five to twelve years**. 5 years (61-71 months), 6 years (72-83 months), 7 years (84-95 months), 8 years (96-107 months), 9 years (108-119 months), 10 years (120-131 months), 11 years (132-143 months) and 12 years (144-155 months).

**Figure 3:**
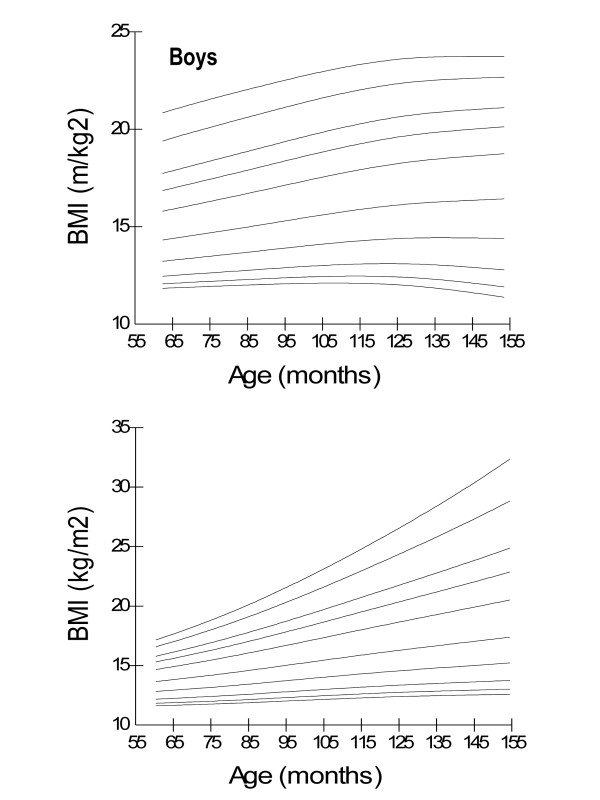
**Smoothed BMI percentile curves for Pakistani primary school boys (n = 997) and girls (n = 883) aged five to twelve years**. 5 years (61-71 months), 6 years (72-83 months), 7 years (84-95 months), 8 years (96-107 months), 9 years (108-119 months), 10 years (120-131 months), 11 years (132-143 months) and 12 years (144-155 months).

**Figure 4:**
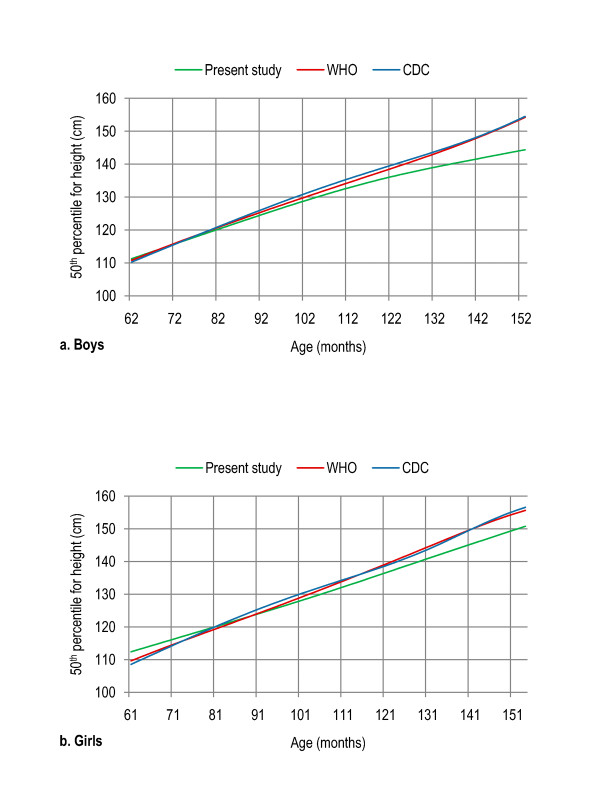
**Comparison of age- and gender-specific 50^th ^height percentile curves for the WHO 2007 reference, USCDC 2000 reference and present study among boys (a) and girls (b) aged five to twelve years**. 5 years (61-71 months), 6 years (72-83 months), 7 years (84-95 months), 8 years (96-107 months), 9 years (108-119 months), 10 years (120-131 months), 11 years (132-143 months) and 12 years (144-155 months).

**Figure 5:**
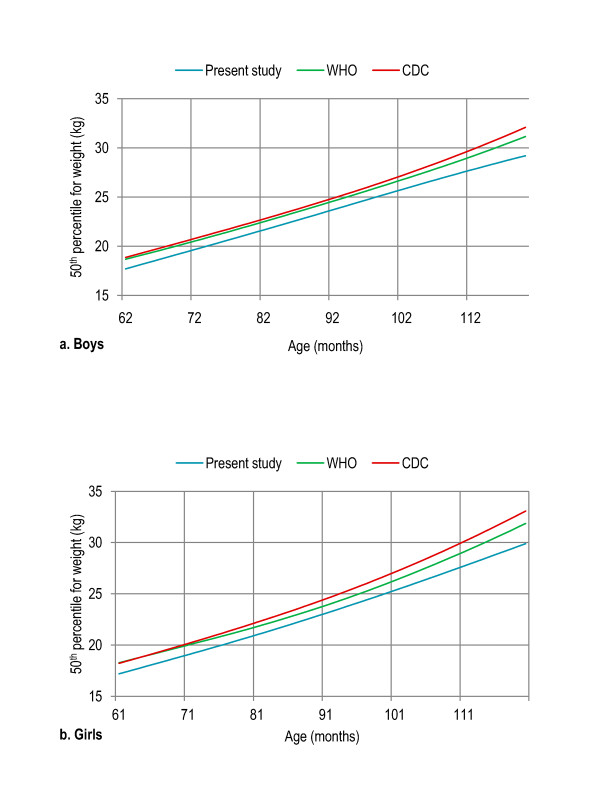
**Comparison of age- and gender-specific 50^th ^weight percentile curves for the WHO 2007 reference, USCDC 2000 reference and present study among boys (a) and girls (b) aged five to twelve years**. 5 years (61-71 months), 6 years (72-83 months), 7 years (84-95 months), 8 years (96-107 months), 9 years (108-119 months), 10 years (120-131 months), 11 years (132-143 months) and 12 years (144-155 months).

**Figure 6:**
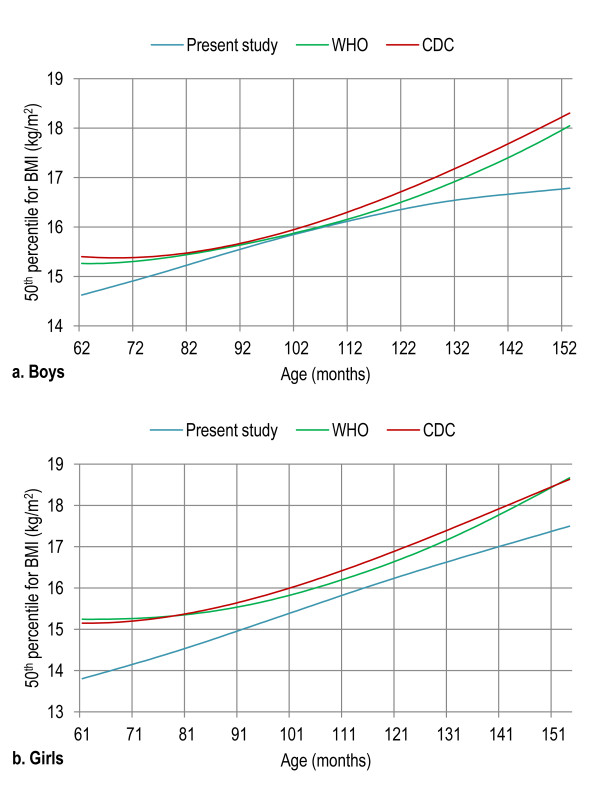
**Comparison of age- and gender-specific 50^th ^BMI percentile curves for the WHO 2007 reference, USCDC 2000 reference and present study among boys (a) and girls (b) aged five to twelve years**. 5 years (61-71 months), 6 years (72-83 months), 7 years (84-95 months), 8 years (96-107 months), 9 years (108-119 months), 10 years (120-131 months), 11 years (132-143 months) and 12 years (144-155 months).

Age- and gender-specific height percentiles (Table 2, Figure 1), age- and gender-specific weight percentiles (Table 3, Figure 2), and age- and gender-specific BMI percentiles (Table 4, Figure 3) were developed and smoothed by the LMS method. Among both boys and girls, height, weight and BMI increased with age. Fiftieth percentile curves for height, weight and BMI of the present sample were compared with the international growth references, the WHO 2007 and USCDC 2000. In younger age groups, boys had approximately the same height while girls were taller; and after nine years of age both boys and girls fell short as compared to the WHO and USCDC references (Figure 4). Both boys and girls had lower weight (Figure 5), and lower BMI (Figure 6), as compared to the WHO and USCDC references.

**Table 2:** Age- and gender-specific smoothed height percentiles for Pakistani primary school children aged five to twelve years (n = 1860)

Characteristics	Percentiles								
	
	3^rd^	5^th^	10^th^	25^th^	50^th^	75^th^	90^th^	95^th^	97^th^
**Boys (n = 977)**									

5 years (61-71 months)	101.5	102.9	105.2	109.0	113.3	117.6	121.5	123.8	125.3

6 years (72-83 months)	105.8	107.4	109.7	113.7	118.2	122.7	126.9	129.3	131.0

7 years (84-95 months)	110.6	112.2	114.6	118.8	123.5	128.3	132.7	135.3	137.1

8 years (96-107 months)	115.1	116.8	119.3	123.7	128.6	133.7	138.3	141.1	143.0

9 years (108-119 months)	119.2	120.9	123.6	128.1	133.3	138.5	143.4	146.4	148.4

10 years (120-131 months)	122.8	124.5	127.2	131.9	137.2	142.7	147.8	150.9	153.0

11 years (132-143 months)	125.7	127.5	130.2	135.0	140.5	146.2	151.5	154.8	156.9

12 years (144-155 months)	128.2	130.0	132.8	137.6	143.2	149.1	154.6	158.0	160.3

**Girls (n = 883)**									

5 years (61-71 months)	102.9	104.2	106.3	109.9	114.1	118.6	122.9	125.6	127.4

6 years (72-83 months)	106.8	108.2	110.4	114.3	118.7	123.5	127.9	130.7	132.5

7 years (84-95 months)	110.7	112.2	114.6	118.7	123.4	128.3	133.0	135.8	137.7

8 years (96-107 months)	114.7	116.3	118.9	123.2	128.2	133.4	138.2	141.1	143.0

9 years (108-119 months)	118.8	120.6	123.3	128.0	133.3	138.7	143.6	146.6	148.6

10 years (120-131 months)	123.1	125.0	127.9	132.9	138.5	144.1	149.2	152.3	154.3

11 years (132-143 months)	127.3	129.3	132.5	137.8	143.7	149.6	154.9	158.0	160.1

12 years (144-155 months)	131.2	133.4	136.8	142.4	148.6	154.8	160.2	163.4	165.5

**Table 3:** Age- and gender-specific smoothed weight percentiles for Pakistani primary school children aged five to twelve years (n = 1860)

Characteristics	Percentiles								
	
	3^rd^	5^th^	10^th^	25^th^	50^th^	75^th^	90^th^	95^th^	97^th^
**Boys (n = 977)**									

5 years (61-71 months)	14.0	14.4	15.1	16.6	18.6	21.1	24.2	26.5	28.3

6 years (72-83 months)	15.4	15.9	16.8	18.4	20.8	23.8	27.4	30.1	32.2

7 years (84-95 months)	17.0	17.5	18.5	20.5	23.2	26.7	30.9	34.2	36.7

8 years (96-107 months)	18.5	19.2	20.3	22.5	25.6	29.7	34.7	38.5	41.4

9 years (108-119 months)	19.9	20.7	22.0	24.5	28.0	32.7	38.3	42.7	46.2

10 years (120-131 months)	21.1	22.0	23.4	26.2	30.1	35.3	41.7	46.7	50.6

11 years (132-143 months)	22.1	23.0	24.6	27.6	32.0	37.7	44.8	50.3	54.6

12 years (144-155 months)	22.9	23.9	25.5	28.8	33.5	39.8	47.5	53.5	58.2

**Girls (n = 883)**									

5 years (61-71 months)	13.8	14.2	14.9	16.2	18.0	20.3	22.9	24.8	26.2

6 years (72-83 months)	15.2	15.7	16.5	18.1	20.3	23.1	26.5	29.0	31.0

7 years (84-95 months)	16.6	17.2	18.2	20.1	22.8	26.3	30.6	33.8	36.4

8 years (96-107 months)	18.1	18.7	19.9	22.2	25.4	29.8	35.1	39.3	42.6

9 years (108-119 months)	19.6	20.4	21.7	24.4	28.3	33.5	40.1	45.4	49.5

10 years (120-131 months)	21.0	21.9	23.5	26.7	31.2	37.5	45.5	51.9	57.1

11 years (132-143 months)	22.3	23.4	25.2	28.8	34.2	41.5	51.0	58.8	65.1

12 years (144-155 months)	23.5	24.7	26.7	30.8	36.9	45.4	56.5	65.7	73.1

**Table 4:** Age- and gender-specific smoothed BMI percentiles for Pakistani primary school children aged five to twelve years (n = 1860)

Characteristics	Percentiles									
	
	3^rd^	5^th^	10^th^	25^th^	50^th^	75^th^	85^th^	90^th^	95^th^	97^th^
**Boys (n = 977)**										

5 years (61-71 months)	11.7	12.0	12.5	12.5	13.4	14.6	16.1	17.0	17.7	19.6

6 years (72-83 months)	11.7	12.0	12.6	12.6	13.6	14.9	16.5	17.5	18.2	20.2

7 years (84-95 months)	11.8	12.2	12.8	12.8	13.9	15.3	17.0	18.0	18.8	20.8

8 years (96-107 months)	11.8	12.2	12.9	12.9	14.1	15.7	17.5	18.6	19.3	21.4

9 years (108-119 months)	11.8	12.3	13.0	13.0	14.3	16.0	17.9	19.0	19.9	22.0

10 years (120-131 months)	11.7	12.2	13.0	13.0	14.5	16.3	18.3	19.5	20.3	22.5

11 years (132-143 months)	11.5	12.1	13.0	13.0	14.6	16.5	18.6	19.8	20.7	22.8

12 years (144-155 months)	11.1	11.8	12.8	12.8	14.6	16.7	18.9	20.2	21.0	23.1

**Girls (n = 883)**										

5 years (61-71 months)	11.7	11.9	12.3	12.9	13.8	14.8	15.4	15.9	16.7	17.3

6 years (72-83 months)	11.8	12.1	12.5	13.2	14.2	15.4	16.2	16.8	17.8	18.6

7 years (84-95 months)	12.0	12.2	12.7	13.5	14.7	16.1	17.1	17.8	19.1	20.1

8 years (96-107 months)	12.2	12.4	12.9	13.9	15.2	16.9	18.1	19.0	20.6	21.9

9 years (108-119 months)	12.3	12.6	13.2	14.2	15.7	17.7	19.1	20.2	22.2	23.8

10 years (120-131 months)	12.5	12.8	13.4	14.6	16.2	18.5	20.1	21.4	23.8	25.9

11 years (132-143 months)	12.6	12.9	13.6	14.8	16.7	19.2	21.1	22.6	25.5	28.0

12 years (144-155 months)	12.7	13.1	13.8	15.2	17.3	20.3	22.5	24.4	28.1	31.4

Mean differences from zero for height-, weight- and BMI-for-age z score values relative to the WHO and USCDC references were significant (P < 0.001). Means of height-for-age (present study: 0.00, 95% CI -0.05 to 0.05; WHO: -0.19, 95% CI -0.25 to -0.13; USCDC: -0.24, 95% CI -0.30 to -0.18), weight-for-age (present study: 0.00, 95% CI -0.05 to 0.05; WHO: -0.22, 95% CI -0.29 to -0.18; USCDC: -0.48, 95% CI -0.54 to -0.41) and BMI-for-age (present study: 0.00, 95% CI -0.04 to 0.04; WHO: -0.32, 95% CI -0.59 to -0.46; USCDC: -0.53, 95% CI: -0.59 to -0.46) z score values relative to the WHO reference were closer to zero and the present study as compared to the USCDC reference (Table 5, Table 6, Table 7).

**Table 5:** Height-for-age z scores relative to the international growth references for Pakistani primary school children aged five to twelve years

Reference	Height-for-age z scores			
	
	Mean	95% CI	SD	P value^a^
**Boys (n = 977)**				

WHO 2007	-0.25	-0.33 to -0.17	1.31	< 0.001

USCDC 2000	-0.31	-0.90 to -0.22	1.29	< 0.001

Present study	0.00	-0.06 to 0.06	1.00	0.990

**Girls (n = 883)**				

WHO 2007	-0.12	-0.21 to -0.03	1.33	< 0.001

USCDC 2000	-0.16	-0.24 to -0.07	1.29	< 0.001

Present study	0.00	-0.07 to 0.07	1.00	0.996

**Total (n = 1860)**				

WHO 2007	-0.19	-0.25 to -0.13	1.29	< 0.001

USCDC 2000	-0.24	-0.30 to -0.18	1.32	< 0.001

Present study	0.00	-0.05 to 0.05	1.00	0.978

^a ^P values are for mean difference from zero for height-for-age z scores (obtained from the study data) relative to the WHO 2007 and USCDC 2000 references, and present study

**Table 6:** Weight-for-age z scores relative to the international growth references for Pakistani primary school children aged five to twelve years

Reference	Weight-for-age z scores			
	
	Mean	95% CI	SD	P value^a^
**Boys (n = 977)**				

WHO 2007	-0.22^b^	-0.33 to -0.12	1.44	< 0.001

USCDC 2000	-0.51	-0.59 to -0.42	1.40	< 0.001

Present study	0.00	-0.06 to 0.06	1.00	0.988

**Girls (n = 883)**				

WHO 2007	-0.21^c^	-0.31 to -0.11	1.34	< 0.001

USCDC 2000	-0.45	-0.53 to -0.36	1.32	< 0.001

Present study	0.00	-0.07 to 0.07	1.00	0.979

**Total (n = 1860)**				

WHO 2007	-0.22	-0.29 to -0.15	1.36	< 0.001

USCDC 2000	-0.48	-0.54 to -0.41	1.39	< 0.001

Present study	0.00	-0.05 to 0.05	1.00	0.983

^a ^P values are for mean difference from zero for weight-for-age z scores (obtained from the study data) relative to the WHO 2007 and USCDC 2000 references, and present study

^b ^n = 747 (boys aged five to ten years) and^c ^n = 699 (girls aged five to ten years) for weight-for-age z scores relative to the WHO 2007 reference

**Table 7:** BMI-for-age z scores relative to the international growth references for Pakistani primary school children aged five to twelve years

Reference	BMI-for-age z scores			
	
	Mean	95% CI	SD	P value^a^
**Boys (n = 977)**				

WHO 2007	-0.30	-0.41 to -0.20	1.60	< 0.001

USCDC 2000	-0.51	-0.61 to -0.42	2.00	< 0.001

Present study	0.00	-0.05 to 0.07	1.00	0.803

**Girls (n = 883)**				

WHO 2007	-0.34	-0.43 to -0.25	1.40	< 0.001

USCDC 2000	-0.54	-0.63 to -0.44	1.44	< 0.001

Present study	0.00	-0.07 to 0.07	1.00	0.999

**Total (n = 1860)**				

WHO 2007	-0.32	-0.39 to -0.25	1.51	< 0.001

USCDC 2000	-0.53	-0.59 to -0.46	1.48	< 0.001

Present study	0.00	-0.04 to 0.04	0.99	0.859

^a ^P values are for mean difference from zero for BMI-for-age z scores (obtained from the study data) relative to the WHO 2007 and USCDC 2000 references, and present study

Differences in means of z score values for weight-for-age (boys: 0.23, 95% CI 0.09-0.36, P = 0.001; girls: 0.17, 95% CI 0.03 to 0.31, P = 0.020; total: 0.19, 95% CI 0.10-0.30, P < 0.001) and BMI-for-age (boys: 0.21, 95% CI 0.07-0.35, P = 0.003; girls: 0.20, 95% CI 0.07-0.34, P = 0.004; total: 0.21, 95% CI 0.11-0.30, P < 0.001) relative to the WHO and USCDC references were significant. Difference in means of z score values for height-for-age relative to the WHO and USCDC references was not significant (boys: 0.05, 95% CI -0.16 to 0.05; girls: 0.04, 95% CI -0.17 to 0.08; total: 0.05, 95% CI -0.03 to 0.13).

Over-nutrition estimates were significantly higher (P < 0.001) by the WHO reference as compared to the USCDC reference (17% vs. 15% overweight and 7.5% vs. 4% obesity). The same trend was observed among both boys (17% vs. 14% overweight and 9% vs. 5% obesity) and girls (16.5% vs. 15% overweight and 6% vs. 3% obesity) (Table 8). Estimates for underweight and thinness/wasting were significantly lower (P < 0.001) by the WHO reference as compared to the USCDC reference (7% vs. 12% underweight and 10% vs. 13% thinness). The same trend was observed among both among boys (7% vs. 13% underweight and 10% vs. 13% thinness) and girls (6% vs. 12% underweight and 10% vs. 13.5% thinness) (Table 9). Difference in stunting prevalence was not significant between the WHO and USCDC references among boys and girls. When the IOTF cut-offs were used, overweight and obesity prevalence was 8% and 5% respectively (8% and 4% respectively among boys and 9% and 5% respectively among girls). Thinness prevalence was 19% (grade one), 6% (grade two) and 4% (grade three). Thinness among boys was 18% (grade one), 5% (grade two) and 4% (grade three) while among girls it was 19% (grade one), 8% (grade two) and 3.5% (grade three). Overweight was significantly lower with use of the IOTF cut-offs as compared to the WHO (P < 0.001) and USCDC (P < 0.001) references. Obesity prevalence was also significantly lower with use of the IOTF cut-offs as compared to the WHO (P < 0.001) and USCDC (P = 0.017) references. Significantly higher thinness (grade one) prevalence was seen with use of the IOTF cut-offs as compared to the WHO (P < 0.001) and USCDC (P < 0.001) references.

**Table 8:** Prevalence of over-nutrition relative to the international growth references among Pakistani primary school children aged five to twelve years (n = 1860)

Reference	Overweight > + 1 SD BMI-for-age z score		Obesity > + 2 SD BMI-for-age z score	
	
	n (%)	P value^a^	n (%)	P value^a^
**Boys (n = 977)**				

WHO 2007	170 (17.4)	< 0.001	86 (8.8)	< 0.001
			
USCDC 2000	141 (14.4)		45 (4.6)	

IOTF^b^	74 (7.6)	< 0.001^c^	42 (4.3)	< 0.001^c^
		< 0.001^d^		0.453^d^

**Girls (n = 883)**				

WHO 2007	146 (16.5)	0.006	54 (6.1)	< 0.001
			
USCDC 2000	135 (15.3)		30 (3.4)	

IOTF^b^	80 (9.1)	< 0.001^c^	45 (5.1)	0.422^c^
		< 0.001^d^		< 0.001^d^

**Total (n = 1860)**				

WHO 2007	316 (17.0)	< 0.001	140 (7.5)	< 0.001
			
USCDC 2000	276 (14.8)		75 (4.0)	

IOTF^b^	154 (8.3)	< 0.001^c^	87 (4.7)	< 0.001^c^
		< 0.001^d^		0.017^d^

^a ^P values are for difference between the estimates by the WHO 2007 and USCDC 2000 references obtained by the McNemar test

^b ^IOTF estimates are based on cut-offs corresponding to the respective BMI cut-off value at age 18 years and above

^c ^compared to the WHO 2007 reference,^d ^compared to the USCDC 2000 reference

**Table 9:** Prevalence of under-nutrition relative to the international growth references among Pakistani primary school children aged five to twelve years

Reference	Stunting < -2 SD height-for-age z score		Underweight < -2 SD weight-for-age z score		Thinness/Wasting < -2 SD BMI-for-age z score	
	
	n (%)	P value^a^	n (%)	P value^a^	n (%)	P value^a^
**Boys (n = 977)**						

WHO 2007	84 (8.6)	0.146	69 (7.1)^b^	< 0.001	99 (10.1)	< 0.001
					
USCDC 2000	90 (9.2)		126 (12.9)		125 (12.8)	

IOTF^c^	-		-		36 (3.7)^d^	< 0.001^g^

					45 (4.6)^e^	< 0.001^h^

					180 (18.4)^f^	

**Girls (n = 883)**						

WHO 2007	68 (7.7)	0.774	56 (6.3)^i^	< 0.001	89 (10.1)	< 0.001
					
USCDC 2000	70 (7.9)		104 (11.8)		119 (13.5)	

IOTF^c^	-		-		31 (3.5)^d^	< 0.001^g^

					70 (7.9)^e^	0.003^h^

					166 (18.8)^f^	

**Total (n = 1860)**						

WHO 2007	152 (8.2)	0.169	125 (6.7)^j^	< 0.001	188 (10.1)	< 0.001
					
USCDC 2000	160 (8.6)		230 (12.4)		244 (13.1)	

IOTF^c^	-		-		67 (3.6)^d^	< 0.001^g^
					115 (6.2)^e^	< 0.001^h^
					346 (18.6)^f^	

^a ^P values are for difference between the estimates by the WHO 2007 and USCDC 2000 references obtained by the McNemar test

^b ^n = 747 (boys aged five to ten years) for underweight prevalence with the WHO 2007 reference

^c ^IOTF estimates are based on cut-offs corresponding to the respective BMI cut-off value at age 18 years and above

^d ^Thinness grade three,^e ^Thinness grade two, and^f ^Thinness grade one

^g ^grade one thinness compared to the WHO 2007 reference,^h ^grade one thinness compared to the USCDC 2000 reference

^i ^n = 699 (girls aged five to ten years), and^j ^n = 1446 (children aged five to ten years) for underweight prevalence with the WHO 2007 reference

## Discussion

Child under-nutrition is estimated to be the largest contributor to global burden of disease, killing millions of children and causing heavy health expenditures in the developing countries particularly South Asia [21-25]. Childhood obesity is a global epidemic that is now penetrating the developing countries including Pakistan, especially amongst the urban [25-29]. Interpretation of child growth in a population depends primarily on the growth reference used, and literature lacks data on indices of nutritional status with reference to the WHO and USCDC references that are potentially useful for Pakistani children [9-11]. The present study is first to report comparison of international growth references among Pakistani school-aged children.

Height, weight and BMI increased with age among both boys and girls. Girls showed a sharp increase in weight and BMI after nine years of age possibly because of pubertal growth spurt that is more pronounced in girls in this age group. Both boys and girls had approximately the same height and lower weight and BMI as compared to the WHO and USCDC references. Previous studies in various countries reported a significant difference of the native children with the WHO and USCDC samples and development of a nationally representative growth reference was suggested for each country [30-33]. Lower BMI cut-off points may need to be set for Asian populations, due to their predisposition to obesity [34-38].

Mean differences from zero for height-, weight- and BMI-for-age z scores relative to the WHO and USCDC references were significant; however, z score means relative to the WHO reference were closer to zero and the present study as compared to the USCDC reference. Mean differences between weight- and BMI-for-age z scores relative to the WHO and USCDC references were significant. Overweight and obesity were significantly higher by the WHO reference as compared to the USCDC reference while underweight and thinness/wasting were significantly lower by the WHO reference as compared to the USCDC reference. Difference between the WHO and USCDC height-for-age z score means and estimates for stunting by the WHO and USCDC references was not significant. Previous studies reported a lower prevalence of underweight and stunting while a higher prevalence of overweight and obesity using the WHO reference compared to the USCDC reference [39-42]. The USCDC growth charts over-estimate the under-nutrition and under-estimate the over-nutrition. The WHO reference has been recommended as a better choice as compared to the USCDC reference and IOTF cut-offs for assessing child growth in the developing countries [39-44]. Significantly higher thinness prevalence and lower overweight and obesity prevalence were seen with use of the IOTF cut-offs as compared to the WHO and USCDC references, consistent with previous studies [45,46].

Prevalence of overweight, obesity, stunting and thinness showed a significantly increasing trend with age and grade [47,48]. Significant correlates of overweight and obesity included urban area with high socioeconomic status (SES), higher parental education and residence in high-income neighborhoods while stunting and thinness were significantly associated with rural area and urban area with low SES, lower parental education and residence in low-income neighborhoods [47-49].

## Conclusions

Pakistani school-aged children significantly differed from the WHO and USCDC references. However, z score means relative to the WHO reference were closer to zero and the present study as compared to the USCDC reference. Overweight and obesity were significantly higher while underweight and thinness/wasting were significantly lower relative to the WHO reference as compared to the USCDC reference and the IOTF cut-offs. New growth charts for Pakistani children based on a nationally representative sample should be developed. Nevertheless, shifting to use of the 2007 WHO child growth reference might have important implications for child health programs and primary care pediatric clinics.

## Competing interests

The authors declare that they have no competing interests.

## Authors' contributions

MUM, principal investigator, conceived and implemented the study, analyzed and interpreted the data, prepared the manuscript and supervised the entire project. SG and KM contributed to the study analysis, interpretation and manuscript preparation. HMA and UK participated in the study implementation and manuscript preparation. US contributed to the study conception, implementation and analysis. MAS and JA oversaw the study conception, implementation and manuscript preparation. All authors read and approved the final manuscript.

## Pre-publication history

The pre-publication history for this paper can be accessed here:

http://www.biomedcentral.com/1471-2431/12/31/prepub
